# Family Planning Knowledge and Practice among People Living with HIV in Nepal

**DOI:** 10.1371/journal.pone.0088663

**Published:** 2014-02-13

**Authors:** Shiva Raj Mishra, Mahesh Prasad Joshi, Vishnu Khanal

**Affiliations:** 1 Institute of Medicine, Maharajgunj Medical Campus, Kathmandu, Nepal; 2 Naulo Ghumti Nepal, Kaski, Nepal; 3 Curtin University, School of Public Health, Perth, Australia; Alberta Provincial Laboratory for Public Health/University of Alberta, Canada

## Abstract

Unsafe sexual behavior is common among the HIV infected. This exposes them to the risks of unintended pregnancy, HIV transmission to uninfected partners and super-infection. Studies on the use of family planning measures among People Living with HIV (PLHIV) are scarce in Nepal. The aim of this study was to explore the knowledge and practice of family planning (FP) in PLHIV. A cross sectional survey was conducted during July–December 2012 in Kaski district of Nepal. A total of 120 PLHIVs were recruited using snowball sampling from three HIV clinics within the Pokhara sub-metropolitan city area. This study found that nine in ten PLHIV had heard about family planning. Two thirds of respondents were using at least one FP method. The majority (65.8%) used condoms and had received FP counseling (67.5%). Less than one percent used condoms in addition to another contraceptive. Being single, being female and having received the counselling sessions were associated with the use of FP. The individuals who received FP counseling were more likely [OR 4.522; 95% CI (1.410–14.504)] to use FP. Females were more likely [OR 4.808; 95% CI (1.396–16.556)] to use FP than males. The individuals who were single/de-facto widowed were more likely [OR 7.330; 95% CI (2.064–26.028)] to use FP than the married individuals. Our findings suggest that there is a need to focus on FP counseling if the HIV prevention program is to increase FP use among the PLHIV population. Use of dual contraceptives need to be promoted through counseling sessions and other health promotion programs focusing in HIV prevention.

## Background

Globally, there are an estimated 33.4 million people living with Human Immunodeficiency Virus/Acquired Immune Deficiency Syndrome (HIV/AIDS) [1]. A significant proportion of people living with HIV (PLHIV) are in Asia, particularly in the Indian subcontinent [2].

Nepal saw the first case of HIV in 1988 and within the period of two decades, it evolved from a mere scattered disease to a concentrated epidemic amongst the population practicing high risk sexual and injecting behaviors [3]. The population of Nepal was 26.6 million in 2012. It has grown by more than three million since 2001 and has nearly doubled in the past 25 years. Large and growing cohorts are entering into their reproductive years and the need for reproductive health services is increasing. The use of contraceptives in Nepal has increased more than ten-fold over the past 30 years– from 3.0 percent in 1976 to 43 percent in 2011 with noted decline between 2006 and 2011 [4] [5], [6]. Government health facilities are the major (69%) providers of FP services in Nepal [5].

Stigma associated with HIV status and discrimination against PLHIV is still prevalent in Nepal in both urban and rural settings [7]. PLHIV face societal stigma, fear of disclosure and discrimination in accessing health facilities: long waiting hours and a lack of awareness about where to seek services [7]. Discrimination may contribute to an increase in HIV-related risk behaviors among PLHIV, such non-use of condoms, non-use of available facilities for CD4 testing and non-use of anti-retroviral therapy (ART) [8]–[12].

PLHIV have fertility desires [13]–[15]. However, this group has a higher number of unplanned pregnancies and can experience negative health effects of pregnancy, leading to poor obstetric outcomes and rapid progression of HIV [16]. The unprotected sexual contact among seroconcordant or disconcordants partners has serious health implications for both themselves and their partners [17]. This increases the chance of HIV infection among serodisconcordants. For those who already have HIV, there is the risk of super infection including drug-resistant strain of HIV and the spread of other Sexually Transmitted Infections (STIs) and diseases [18] [19]. In addition, this contributes to HIV infections among newborns through vertical transmission. Thus, there is a cycle of unregulated fertility, HIV re-infection and suffering among PLHIV. In this scenario reducing the chance of unintended pregnancy and risk of HIV transmission can be achieved by providing comprehensive FP services through dual FP; condom and other methods [20].

Health workers’ attitude and misconceptions about the appropriateness of contraceptive methods for PLHIV constitute a challenge to FP service delivery [21], [22]. This has resulted in PLHIV undergoing abortion and high rates mother to child transmission. As of 2012, the total number of children under five living with HIV as a result of mother to child transmission was 1083 in Nepal (6). With the start of prevention of mother to child transmission of HIV in 2005, the number of women who are tested for HIV during their attendance of antenatal clinics is increasing [3]. The 2011 Annual report from the Ministry of Health, Nepal showed that 93 out of 66926 women were HIV positive during such screening [3]. Due to poor surveillance and lack of testing services in the vast majority of antenatal clinics, the current data only represents hospitals where such testing facilities are available. Therefore, the current status may be only the tip of iceberg. A separate figure of pregnancy and childbearing among PLHIV is not available.

The use of condoms along with other forms of contraceptive- dual method- is the recommended contraceptive measure for PLHIV when the chances of unintended pregnancy are high [20]. A study in India found that condoms were the most common contraceptive method amongst married men and women living with HIV [23], [24]. The use of dual-contraceptive in India was only 5% before HIV diagnosis and 23% after diagnosis [23]. Another study from India reported inconsistent condom use among PLHIVs [24]. This stressed the promotion of dual FP, male involvement in FP, provision of comprehensive FP information, clarification on misconception during counseling and need for national guidelines for FP/HIV integration [23]. In a study in Kathmandu Valley, 75% of HIV positive men had sex in the past 6 months and only 46% used condom regularly [25]. Another study from Nepal reported that 50.6% of HIV positive men intended to practice safer sex every time they had sex with seroconcordant partners [17]. In Nepal, there is little evidence on the use of FP methods among the PLHIV. Updated knowledge and evidence on this subject will help the policy makers and program managers to design, justify and implement HIV prevention programs and FP programs in Nepal. The aim of this study was to explore FP practice among PLHIV in Nepal.

## Methods

### Study Setting

This study was conducted in the Kaski district of the Western Region of Nepal. The Kaski district has a population of 492,098 people, of which 53% (264,991) are living in Pokhara sub-metropolitan city [26]. The Kaski District AIDS Coordination Committee has estimated that there are 1,178 HIV positive cases in the district. A total of 1015 persons aged 15 years and over are receiving treatment, care and support [27].

Naulo Ghumti Nepal- an NGO based in Kaski district- provides community and home based care to PLHIV in the area. Likewise, a public hospital- the Western Regional Hospital- offers CD4 testing and treatment of opportunistic infections. Likewise, residential care and support is provided by Community Support Group, Kaski.

### Ethical Approval

This study protocol was approved by the Human Research Ethics Committee, Pokhara University, Kaski, Nepal. Written informed consent was taken from participants before the start of each interview. Researchers recorded no personal identifiers to ensure confidentiality of participants. Additionally, participants were not given any compensation for participating in this research.

### Study Design and Sampling

This cross sectional survey was conducted between July and December 2012. Respondents were recruited by snowballing from three HIV clinics within the Pokhara sub-metropolitan city area [28], [29]. Maximum care was given to ensure recruitment of diverse respondents by age, ethnicity, and education. The total sample size of 120 was calculated at a 95% confidence interval taking the premise that p = 0.43 (national prevalence of FP ), the allowable error (0.1) is 10% and the non-response rate 10% [30].

The inclusion criteria for this study were: (i) HIV-infected women and men aged 15–49 years, (ii) attending outpatient HIV clinics in recruiting sites, and (iii) giving a written consent to participate in the study. Three trained enumerators were recruited and trained for data collection in this study. They participated in sampling frame generation and the initial coordination exercise with the organization working with PLHIV. By doing this, they became acquainted with the research objectives and data collection tools.

### Data Collection Procedures

Face to face interviews were carried out to collect socio-demographic information, sexual and reproductive history, FP knowledge and use, fertility desires and intentions and experiences of stigma. The questionnaire on womens’ and mens’ contraceptive use was adapted from the 2006 Nepal Demographic and Health Survey (NDHS) and a similar study from Uganda [31], [32]. The questionnaire was translated into Nepali and back translated into English. The Nepali version of the questionnaire was pretested among the PLHIVs visiting HIV clinics in Naulo Ghumti Nepal, Kaski, Nepal.

### Definition of Variable

#### Outcome variable

Knowledge about FP was assessed by asking five general statements and ten method specific statements (yes/no).

1. FP is a method to prevent unwanted birth
2. FP is a method to bring wanted birth
3. FP is a method to maintain gap between two births
4. FP is a method to maintain happy & healthy family
5. All of the above.

Currently used FP methods were assessed by direct questioning. Reasons for not using any FP methods were ascertained.

#### Explanatory variable

The socio-demographic information included in this study were age, gender, school education, ethnicity, religion and occupational status (employed versus unemployed). Ethnicity was categorized as advantaged and disadvantaged ethnic groups based on the Health Management Information System classification and similar studies in Nepal [33]. The advantaged ethnic group includes the upper caste group and relatively advantaged Janjati, while the disadvantaged ethnic group includes Dalit, disadvantaged Janjati; and,disadvantaged non- Dalit includes Terai caste groups and religious minorities. Religion included Hindu and Non-Hindu and occupational status as employed (currently working in a paid job) and unemployed. Participants were asked which sources information about FP had obtained from, including radio, TV, and newspapers. Respondents were asked whether they helped their families with their earnings in the last month. Respondents relationships with their family were categorized as very good (4 to 5), moderate (2 to 3), not-good at all (1) by simply asking respondents to rate their family relationship on a 1 to 5 Likert scale.

### Data Analysis

Socio-demographic characteristics and the reproductive and HIV related behaviors and practices were summarized in percentages. Association between FP use and other independent variables were analyzed by using a chi-square test. Variables which were significantly associated at the p<0.10 level in chi-square test were evaluated in multivariate logistic analysis. A p-value <0.05 was considered statistically significant in multivariable analysis. Data analysis was performed by using Statistical Package for Social Sciences (SPSS) Statistics Version 19 for Windows (SPSS Inc., Chicago, Illinois, USA).

## Results

### Demographic Findings

Table 1 presents the characteristics of the participants. This study recruited 64 male and 56 female respondents. Mean age of respondents was 35.1 years (± SD 7.32). Adults of age group 25–49 covered the highest portion of respondents - 94.2% (mean 35.06 years). Among the respondents, 70.8% were Hindu, 24.2% were illiterate, 77.5% were married, and 20.8% were unemployed. Slightly more than half of the respondents were from disadvantaged group (55.0%).

**Table 1:** Demographic findings.

Characteristics	Total n (%)	Male n (%)	Female n (%)
**Age** (35.06±7.32) (n = 120)			
Adolescents (11–19)	1(0.83)	0 (0)	1(0.83)
Early adults (20–24)	6(5.00)	2 (1.67)	4(3.33)
Adults (25–49)	113(94.17)	62 (51.67)	51(42.5)
**Religion** (n = 120)			
Hindu	85(70.83)	45(37.5)	40(33.33)
Others (Christian, Buddhism, Muslim, Kirat)	35(28.46)	19(15.83)	16(13.33)
**Ethnicity** (n = 120)			
Advantaged group	54 (44.30%)	31 (25.83%)	23 (19.17%)
Disadvantaged group	66(55.00%)	33 (27.50%)	33 (28.50%)
**Education**(n = 120)			
Illiterate	25(20.83)	10(8.33)	15(12.5)
Primary	27(22.5)	15(12.5)	12(10)
Lower secondary	28(31.67)	23(19.17)	15(12.5)
Higher Secondary & Above	30(25)	16(13.33)	14(11.67)
**Marital Status**(n = 120)			
Married	93(77.5)	54(45)	39(32.5)
Unmarried	16(13.33)	6(5)	10(8.33)
Others	11(9.16)	4(3.33)	7(5.83)
**Occupation**(n = 120)			
Agriculture/household work	22(18.33)	10(8.33)	12(10)
Public Service	23(12.5)	8(6.67)	15(12.5)
Business	18(15)	12(10)	6(5)
Labor/Transportation/Agriculture	28(9.33)	19(1.83)	9(7.5)
Others	29(24.17)	15(12.5)	14(11.67)

### Knowledge Level among Respondent

Table 2 presents the knowledge on FP among the respondents. The majority (93.3%) of respondents had heard of FP. The majority (53.6%) said that FP is a method to prevent unwanted birth, followed by it brings wanted birth (24.1%), maintains gap between two births (14.3%), and maintains happy and healthy family (4.5%). In total, only 5.0% could tell all four meanings of FP. Health institutions were the major source (54.2%) of FP related information. Almost all (98.3%) had heard of condoms as a temporary method of FP and only 55.8% had heard of male sterilisation as a permanent method.

**Table 2:** FP related information.

Statements	Yes n (%)	No n (%)
FP knowledge		
**Have you ever heard about family planning?**	112 (93.3)	8(6.7%)
FP is methods to prevent unwanted birth	60(53.57)	52(46.43%)
FP is methods s to bring wanted birth	27 (24.11)	85(75.89)
FP is methods to maintain gap between 2 births	16 (14.29)	96(85.71)
FP is methods to maintain happy & healthy family	5(4.46)	107(95.54%)
All of the Above	6(5.36)	106(94.64%)
**Sources of FP related information**		
Health institution	65 (54.2%)	55(45.8%)
Family/friends	29(24.2%)	91(75.8%)
School/college	11(9.2%)	109(90.8%)
Newspaper/radio/TV (electronic media)	58(48.3%)	62(51.7%)
**Heard about FP methods**		
Heard about natural method Abstinence	24(20%)	96(80%)
Heard about natural method Withdrawal method	28(23.3)	92(76.7)
Heard about natural method Calendar method	11(9.2%)	109(90.8)
Heard about temporary method Condom	118(98.3)	2(1.7)
Heard about temporary method Pills	69(57.5)	51(42.5)
Heard about temporary method Depo(DMPA)	60(50)	60(50)
Heard about temporary method Copper T (IUCD)	49(40.8)	71(59.2)
Heard about temporary method Implant	48(40)	72(60)
Heard about permanent method Male sterilization	67(55.8)	53(44.2)
Heard about permanent method Female sterilization	55(45.8)	65(54.2)
**Practice Statements**		
Are you currently using any family planning method?	85 (70.8)	35(29.2)
Currently using Male Condom	79(65.8)	41(34.2)
Currently using temporary method Pills	3(2.5)	117(97.5)
Currently using temporary method Depo (DMPA)	1(0.8)	119(99.2)
Currently using permanent method	3(2.5)	117(97.5)
Did you used condom in last 3 months?	70(58.3)	50(41.7)
Did you use condom every time you had sex?	88(73.3)	32(26.7)
Have you ever received FP counseling in health center?	81(67.5)	39(32.5)
Have you ever used FP services from health center?	63(52.5)	57(47.5)

### Use of FP among the Respondents

Table 2 presents the use of FP among the respondents. The majority (70.8%) were using at least one FP method. Two thirds (65.8%) used condoms and only 2.5% used the oral contraceptive pill. The majority (67.5%) of respondents had ever received FP counseling. Current study showed that only 0.8% simultaneously used condoms and another FP method.

When asked of reasons for not using FP services, lack of knowledge about availability of FP services (20.0%), lack of resources to access (8.6%) and fear of stigma and discrimination (14.3%) were the major ones mentioned. The other reasons for not using FP services were having irregular sexual partners, being unmarried/single and illness. Surprisingly, travelling distance to health centers was not reported as a reason for non-use (not shown in Table).

### Factors Associated with the use of FP

Sex, marital status, family relationship, helping family from what he/she earns, regular sexual intercourse, having received FP counseling and attitude variables like believing that FP is useful to prevent unwanted pregnancy and that FP can be used before marriage were found significant in Chi square test (Table 3). Variables significant in chi-square test were entered into logistic regression analysis. Sex of the respondent, marital status, having regular sexual intercourse and receiving FP counseling were significantly associated with the use of FP in multiple logistic regression analysis. Females were more likely [OR 4.808; 95% CI (1.396–16.556)] to use FP than males. The individuals who were single/de-facto widowed were more likely [OR 7.330; 95% CI (2.064–26.028)] to use FP than the married individuals. The individuals who received FP counseling were more likely [OR 4.522; 95% CI (1.410–14.504)] to use FP. The individuals who had regular sexual intercourse were less likely [OR 0.072; 95% CI (0.015–0.356)] to use FP.

**Table 3:** Factors associated with current family planning use.

		Currently using contraceptives		aOR (95%CI)	P-value
Variable	Total (n)	Yes n(%)	No n(%)		
**Age group**					
15–29 years	32	26(81.3%)	6(18.8%)		
30–49 years	88	59(67%)	29(33%)		
**Sex**					
Female	56	29(51.8%)	27(48.2%)	[4.808; 95% CI (1.396–16.556)]	0.013
Male	64	56(87.5%)	8(12.5%)		
**Religion**					
Hindu	85	61(71.8%)	24(28.2%)		
Other	35	68.6%)	11(31.4%)		
**Educational status**					
Illiterate	25	16 (64%)	9(36%)		
Literate	95	69(72.6%)	26(27.4%)		
**Marital status**					
Single/divorced/widowed	93	77(82.8%)	16(17.2%)	[7.330; 95% CI (2.064–26.028)]	0.002
Married/de facto	27	8(29.6%)	19(70.4%)		
**Occupation**					
Unemployed	29	18(62.1%)	11(37.9%)		
Employed	91	67(73.6%)	24(26.4%)		
**Helps from what he/she earns**					
Yes	90	71(78.9%)	19 (21.1%)		
No	30	14(46.7%)	16(53.3%)		
**Family relationship**					
Good	93	70 (75.3%)	23 (24.7%)		
Moderate	24	15(62.5%)	9(37.5%)		
Worse	3	0(0%)	3(100%)		
**Regular sexual intercourse**					
Yes	54	52(96.3%)	2 (3.7%)	[0.072; 95% CI (0.015–0.356)]	0.001
No	66	33(50%)	33(50%)		
**Number of HIV positive children**					
No children	20	13(65%)	7(35%)		
1 child or more	6	3(50%)	3 (50%)		
**Received FP counseling**					
Yes	81	66 (81.5%)	15(18.5%)	[4.522; 95% CI (1.410–14.504)]	0.011
No	39	19(48.7%)	20(51.3%)		
**On ARV**					
Yes	103	71 (68.9%)	32(31.1%)		
No	17	14 (82.4%)	3 (17.6%)		
**Can we use contraceptives during ARV?**					
Yes	85	65 (76.5%)	20(23.5%)		
No	24	13(54.2%)	11(45.8%)		
Don’t know	11	7(63.6%)	4(36.4%)		
**FP useful to prevent unwanted pregnancy?**					
Strongly agree	34	30(88.2%)	4 (11.8%)		
Agree	77	48(62.3%)	29(37.7%)		
Disagree	1	1(100%)	0(0%)		
Don’t know	8	6(75%)	2(25%)		
**FP can be used before** **marriage?**					
Strongly agree	25	22 (88%)	3 (12%)		
Agree	65	44(67.7%)	21(32.3%)		
Disagree	21	13(61.9%)	8(38.1%)		
Don’t know	9	6(66.7%)	3(33.3%)		
**Heard about positive prevention**					
Yes	72	52(72.2%)	20(27.8%)		
**No**	48	33(68.8%)	15(31.3%)		
**Heard about dual method of protection**					
Yes	63	43(68.3%)	20(31.7%)		
**No**	57	42(73.7%)	15(26.3%)		
**Desire for children**					
**Yes**	14	12(85.7%)	2(14.3%)		
**No**	106	73(68.9%)	33(31.1%)		

## Discussion

The use of FP is important for overall health. It becomes even more important in the case of PLHIV, due to fertility desires, the chance of unplanned pregnancies and the subsequent negative health effects. In Nepal, there is low access to anti-retroviral therapy; therefore, unregulated fertility can lead to higher mother to child transmission, super-infection of HIV and the consequences arising out of these events [18]. So far, there has not been any research to explore FP knowledge and practice among this key population in Nepal. This study reports the knowledge, practice and factors associated with FP use among PLHIV in Nepal.

More than two thirds of the respondents in this study (70.8%) reported having used contraceptive. This is lower than sexually active PLHIV using FP services in Uganda (87.3%) and India (95.0%) [23], [34]. The current study showed that two thirds used condoms and none were using natural methods of FP. Condom use reported in this study is lower than reported among PLHIV in India (92%) [23]. According to a revised report of UNFPA, WHO, UNAIDS in 2009, condom use is the most efficient method to reduce the sexual transmission of HIV and STIs [35]. Condom use needs to be universal as every sexual encounter is at a high risk if it is unprotected and involves an HIV infected person [36], [37].

This study showed that only 0.83% used dual methods of FP. This is low in comparison to other developing countries such as India (23%), Rwanda (1%), and Malawai (1%) [23], [38], [39]. The current lower rate of dual contraception can be explained by two main reasons: first, low emphasis on dual FP during FP counseling sessions; second, misconceptions about non condom FP [23]. In the context of Nepal, whereas FP knowledge is universal among the general population [5],misconceptions about FP is common among PLHIV [40]. Future action should be directed towards motivating PLHIV on using dual FP methods to avoid the risk of HIV, STIs and unplanned pregnancy [41], [42].

Almost all had heard of condoms, whereas the calendar method was the least known. Very few (5%) could state all four uses of FP**.** The knowledge about timing, spacing and limiting role of contraceptive devices was low in this study. This lower level of knowledge could be explained by the fact that a quarter of the respondents were illiterate. The education system does not give attention to sexuality and reproductive education in the curricula [43], [44]. A previous study on HIV also reported a poor knowledge of HIV among Nepalese males [45]. Generally, health institutions and electronic media were the major sources of HIV/FP information. Also, PLHIV acquire this information during ART counseling sessions, community and home based care. Comprehensive FP/HIV information should be provided to PLHIV during visits to health institutions.

There was a difference in knowledge of temporary and permanent methods of FP. Respondents had heard about temporary methods more often than permanent methods. This could be due to a low priority given to the permanent methods of FP during the counseling sessions by health care providers. The FP services provided to the general population by governmental and non-governmental facilities do not specifically target PLHIV in Nepal [3]. There is also lack of systematic integration of FP and HIV services in the HIV clinics [6], [46]. Due to this, these services often miss PLHIV who have an unmet need for FP.

Males were more likely to use any FP methods than females. This is due to large number of study participants relying on male condoms for contraception. This finding may also indicate that males were mainly involved in use or non-use of FP and women have less control over such decisions. This study revealed that the individuals who received FP counseling were more likely to use contraceptives. Counseling empowers and enables participants to make informed decisions. Such informed choice is likely to increase the consistent use of chosen method. Counseling also provides information about the benefits of safer sex, increases self-efficacy, increases negotiation skills with condoms and promotes disclosure of HIV status [24]. The counseling sessions are also deemed to act as tailored session to offer individualized information based on the client’s need. The positive effect of FP counseling on increasing FP service use has been demonstrated in India and Turkey [47], [23] [48]. Therefore, counseling on contraceptive use should be emphasized in national HIV programs. Currently pre-test and post-test counseling is a part of voluntary counseling and testing services in Nepal; these should also include FP counseling as a routine [46], [49].

Remarkably, the individuals who had regular sexual intercourse were less likely to use FP; the current findings might be due to higher number of married individuals. Married individuals tend to not use FP whilst having sex with their partners, perceiving a lower risk. This should be viewed as important, as this will increase risk of HIV transmission to non-infected partners and super-infection among the PLHIV population [18], [19].

The individuals who were married were less likely to use FP than single/defacto/widowed individuals. This implies that single/defacto/windowed was more sexually active and using FP than married respondents. Single/defacto/widowed PLHIV may feel that they are at risk of transmitting HIV virus to their casual sex partner. Based on the Health Belief Model, when people perceive themselves at risk of having an infection, they are more likely to adopt certain behaviors to avoid the risk [50]. This could explain in part why the more single/defacto/widowed were found to have used FP methods in this study. This is in contrary with findings from Uganda where FP use was significantly higher with being married [31]. Further studies should focus on the specific reason for such lower use of contraception among married individuals.

FP and HIV services are high priority programs in Nepal [3]. Despite there being a close relationship between FP programs and HIV and vice versa, the integration of FP and HIV services has not yet been possible [6], [46]. National FP programs do not specifically target hard-to-reach subpopulation like PLHIV [6]. Therefore, the national FP program should also include the issue of the need of FP among PLHIV. This is the first study on the use of FP measure among the PLHIV population; therefore, the information will be useful for the related stakeholders in decision making. A number of limitations need to be considered when interpreting the findings of this study. This was a cross sectional study so causality cannot be established. Most of the participants were associated with PLHIV networks. They might already have been involved in some information sessions; therefore, the higher knowledge on the FP measures such as condoms might be an over estimation. Future studies may focus on how the unmet need of FP among PLHIV can be improved.

## Conclusion

Almost all PLHIV had heard about condom use but less than 1% used dual methods of FP. Three in ten were not using any FP methods. PLHIV who had received counseling had an increased likelihood of FP use. People who were married and males were less likely to use FP methods. An FP counseling component needs to be integrated and emphasized in HIV services. Counseling on dual FP needs to be promoted in community and health facilities. Further FP promotion among PLHIV should focus on individuals who are married and should include females in decision making regarding FP use.
